# Measuring Erythrocyte Sedimentation Rate Using Photometric Rheology Technology Demonstrates Superior Sample Stability as Compared to the Westergren Method

**DOI:** 10.3390/diagnostics16162648

**Published:** 2026-08-20

**Authors:** Thomas Koshy, Yenny Lamazares, Susan Evans, Saeed Jortani

**Affiliations:** 1 Beeatus Solutions, Inc., Palatine, IL 60067, USA; 2 Kentucky Clinical Trials Laboratory, Louisville, KY 40202, USA; ylamazares@kentuckyctl.com; 3 Biodecisions Consulting, Los Gatos, CA 95033, USA; biodecisions@gmail.com; 4 Department of Pathology and Laboratory Medicine, University of Louisville School of Medicine, Louisville, KY 40292, USA; saeed.jortani@louisville.edu

**Keywords:** erythrocyte sedimentation rate (ESR), inflammation, iSED, Westergren method, blood sample stability, rouleaux formation, red blood cell aggregation, photometric rheology

## Abstract

**Background/Objectives**: Erythrocyte sedimentation rate (ESR) is one of the most widely used tests for assessing inflammatory conditions. Current guidelines require that Erythrocyte Sedimentation Rate (ESR) testing be performed within four hours of collection for samples stored at RT or within 24 h if refrigerated; this is challenging with dispersed collection sites. Thus, we investigated whether testing technology affects sample stability by comparing results obtained using iSED^®^, based on photometric rheology, with the classical Westergren method. **Methods**: Samples were evaluated at multiple time intervals. ESR values from each timepoint were plotted against their Time 0 values, and correlation statistics were analyzed using Passing-Bablok regression analysis and a Stability Drift analysis. Samples were considered stable if the correlation statistics of the Passing-Bablok analysis were statistically indistinct from the identity line. **Results**: Samples in the iSED/RT cohort were stable for up to 28 h after collection, while those in the iSED/4–8 °C cohort were stable for up to 48 h. Samples in the Westergren/RT cohort were stable for up to 10 h after collection, but significantly deteriorated at 12 h and beyond. **Conclusions**: The iSED methodology provides accurate ESR results for samples stored up to 28 h at RT and 48 h at 4–8 °C, which was significantly superior to the Westergren method for which samples were stable at RT for only 10 h.

## 1. Introduction

The erythrocyte sedimentation rate (ESR) is one of the oldest and most widely used laboratory tests in the world [1,2]. It is a marker of subclinical, acute, and chronic inflammation and can be used to assist in diagnosing or monitoring patients with inflammatory conditions [3]. ESR has long been used as a “sickness indicator” due to its non-specific nature, accessibility, and low cost [3]. Unlike other laboratory tests that detect or quantify specific analytes, ESR is measuring the behavior of the red blood cells while the sample is being analyzed [4]. Despite the discovery of newer biomarkers, ESR has remained clinically relevant and valuable because it is sensitive to diffuse low grade conditions, remains stable over time, and can provide complementary information when used in conjunction with acute phase proteins like CRP [5,6]. It remains particularly valuable in diagnosing and monitoring autoimmune diseases [7,8], helping detect low-virulence infections [9,10], assessing fever of unknown origin [11,12], and in certain cancer-related situations [13,14].

ESR is based on the principle that red blood cells settle faster when there are increased plasma proteins in the blood due to inflammation. Typically, the negative charge on the surface of RBCs causes the cells to repel each other, but the presence of acute phase plasma proteins neutralizes the negative charge, enabling and promoting RBC aggregation—or rouleaux formation—the first step of RBC sedimentation [15]. The amount of RBC aggregation is proportional to the amount of red cell sedimentation. The Westergren method, developed in 1921 [16], consists of placing anticoagulated blood in a vertical column and measuring how far the cells travel down the column over a period of one hour [17]. The Westergren method remains the internationally recognized reference method [17,18] but has several well-documented limitations. Westergren ESR results can be influenced by the laboratory environment (temperature and humidity, and vibrations), sample age and storage conditions, and sample variables such as hematocrit, red blood cell (RBC) concentrations, and mean corpuscular volume (MCV). Since the Westergren method is a manual test, results are also dependent on operator technique (e.g., sample mixing, tube filling and angle, and operator subjectivity) [19] and require significant hands-on time. One particular limitation, specifically relevant for outpatient settings, is sample stability [19]. Westergren samples must be tested within 4 h of collection if stored or shipped at room temperature (RT) and within 24 h if refrigerated [17,20]. This poses significant challenges when samples must travel between dispersed collection sites and centralized testing locations under unpredictable temperature control [4]. It is worth noting that other common hematology tests—such as complete blood counts (CBCs)—have wider sample stability windows [21], making ESR stability an anomaly among routine hematology laboratory tests.

These limitations contributed to the development of modified Westergren and alternative ESR methods that offer advantages like less hands-on time and operator variability, less subjectivity in determining results, shorter analysis time, and reduced biohazard exposure [22]. In 2017, the ICSH acknowledged the emergence of these new ESR testing methods and provided recommendations for laboratories to ensure that only validated instruments were used [23]. Since research on ESR sample stability was scarce [4] the specifications provided for the Westergren method were applied to these new methods and manufacturers of modified Westergren and alternate ESR methods originally retained the 4 h RT/24 h refrigerated sample stability windows.

To this end, we evaluated whether ESR sample stability is affected by the testing method used because modern ESR technologies measure earlier portions of the red blood cell (RBC) sedimentation process compared to the Westergren method. The current study (Stabil-iSED) directly compared samples analyzed by the Westergren method with the iSED automated ESR analyzer method (ALCOR^®^ Scientific, Smithfield, RI, USA). The latter uses photometric rheology to produce rapid results using capped EDTA-anticoagulated whole blood samples [2,24]. iSED analyzers directly measure RBC aggregation during rouleaux formation, the first step of erythrocyte sedimentation, whereas the Westergren method measures RBC sedimentation after 60 min [16,19]. RBC aggregation is proportional to RBC sedimentation and photometric rheology correlates with Westergren characteristics. iSED analyzers, using a full automated workflow that includes a 3 min sample mixing time, assess RBC aggregation by monitoring light transmission through a flow cell maintained at 37 °C. Importantly, because iSED captures the initial kinetics of rouleaux formation, these ESR results are determined within 20 s [24].

## 2. Materials and Methods

This study’s major objective was to compare the stability of K2 EDTA anticoagulated whole blood samples tested by iSED to the Westergren method when stored at RT and 4–8 °C. The study was reviewed and approved by the WCG IRB (IRB #20244298, approved 26 November 2024) and was conducted according to FDA 21 CFR Parts 50, 54, 56 and 312, the International Council for Harmonisation-Guidelines for Good Clinical Practice, and the Declaration of Helsinki.

Consenting healthy volunteers and patients ≥ 18 years with conditions related to an elevated ESR or inflammation were screened at two hospitals. Critical care patients for whom drawing additional blood samples posed a health risk, or who presented with hematological abnormalities, were excluded. The target sample size was ≥60 samples spanning the ESR analytical range of 1–130 mm/h, with ≥20 samples in each third of the range. This target sample number was based on guidance from the International Council for Standardization in Haematology (ICSH) regarding accuracy for validating ESR methods [23].

ESR testing was performed using the iSED ELITE ESR Analyzer and the Globe Scientific Sedi-Rate Westergren E.S.R. System (Thermo Fisher Scientific, Inc., Waltham, MA, USA). Blood samples were collected into BD Vacutainer EDTA collection tubes (Becton, Dickinson and Company, Franklin Lakes, NJ, USA). iSED analytical performance was monitored using external controls (SEDiTROL^®^ Quality Control, ALCOR Scientific, Smithfield, RI, USA).

After informed consent, blood samples from each individual were drawn into three tubes: one 7 mL tube and two 4 mL tubes. Hemolyzed, grossly lipemic, and visibly clotted samples were excluded. If fewer than three tubes were collected from a given subject, that sample was dedicated to the iSED/RT assessment. Blood from one 4 mL sample was tested with iSED to establish a baseline ESR, with values categorized into one of three ranges of results: (1) ≤40 mm/h, (2) 41–79 mm/h, or (3) ≥80 mm/h, and the tube was maintained at RT (18–25 °C). The other 4 mL sample was stored at 4–8 °C. A baseline Westergren ESR was recorded with blood from the 7 mL sample, which was then maintained at RT. This produced three cohorts: (1) iSED/RT, (2) iSED/4–8 °C and (3) Westergren/RT.

After the baseline ESR determination, the Westergren method was performed after 8, 10, 12, and 24 h (±30 min) using blood from the 7 mL tube. The iSED method was performed after 8, 10, 12, 24, 28, 36 and 48 h (±30 min) for samples stored at RT, and 8, 24, 36, and 48 h (±30 min) for samples stored at 4–8 °C.

All iSED and Westergren tests were conducted according to the manufacturer’s instructions for use [24,25]. Both tests were required to be completed within 60 min of the targeted time. Before testing, the samples stored at 4–8 °C were brought to room temperature for ≥15 min. Westergren samples were mixed on a rocking table for ≥3 min before testing.

Sample stability was assessed using a novel utilization of Passing-Bablok correlation statistics. For all three cohorts, ESR values at each timepoint were compared to their respective baseline values using Passing-Bablok regression analysis. Samples were considered stable at a given timepoint (i.e., statistically indistinct from the baseline value) if the 95% confidence interval (CI) of the slope and intercept included 1.00 and 0.00, respectively, and the Spearman correlation coefficient was ≥0.90. All analyses were performed using statistical software (MedCalc version 22.023; MedCalc Software Ltd., Ostend, Belgium).

Sample stability was also evaluated using a Stability Drift plot comparing results obtained at each storage time point (8, 10, 12, 24, 28, 36, and 48 h) with the corresponding baseline measurement. In this analysis, the mean of the percent differences between each time point and the baseline were plotted against the hours of that time point, thereby providing a temporal assessment of sample stability.

## 3. Results

Sixty-three patients and five volunteers consented and provided samples. Data are shown in Table 1.

Sixteen samples were removed from the Westergren/RT and iSED/4–8 °C cohorts for the following reasons: baseline ESR value was above the upper limit of the iSED test (>130 mm/h), sample volume was inadequate, or the sample would have been allocated to a cohort that already reached its target. Two of those samples had sufficient volume for testing by one method and were used in the iSED/RT cohort only. Three samples in the iSED/RT cohort had an uncharacteristic decline in ESR values between the 12 h and 24 h timepoints. This pattern was not observed at any other timepoints or in any of the other samples or cohorts. An outlier analysis of the percentage difference between the 12 h and 24 h ESR values of the iSED/room temperature cohort identified these three samples as being greater than the upper interquartile fence for this data set (Q3 + 1.5 × IQR). We considered these differences sufficient to justify categorizing the three samples as outliers and therefore removed them from the regression analyses. Sample categorization based on ESR values for each cohort is shown in Table 2.

Regression analysis results for sample stability are shown in Table 3. Samples in the iSED/RT cohort were stable at timepoints up to 28 h after collection, and those in the iSED/4–8 °C cohort were stable up to 48 h. Samples in the Westergren/RT cohort were stable up to 10 h after collection.

The iSED/RT and iSED/4–8 ° C results at the 8 h timepoints met the slope and coefficient criteria but did not meet the intercept criteria because the CIs of their *y*-axis intercepts did not include 0.00. This is addressed in Section 4.

**Figure 1:**
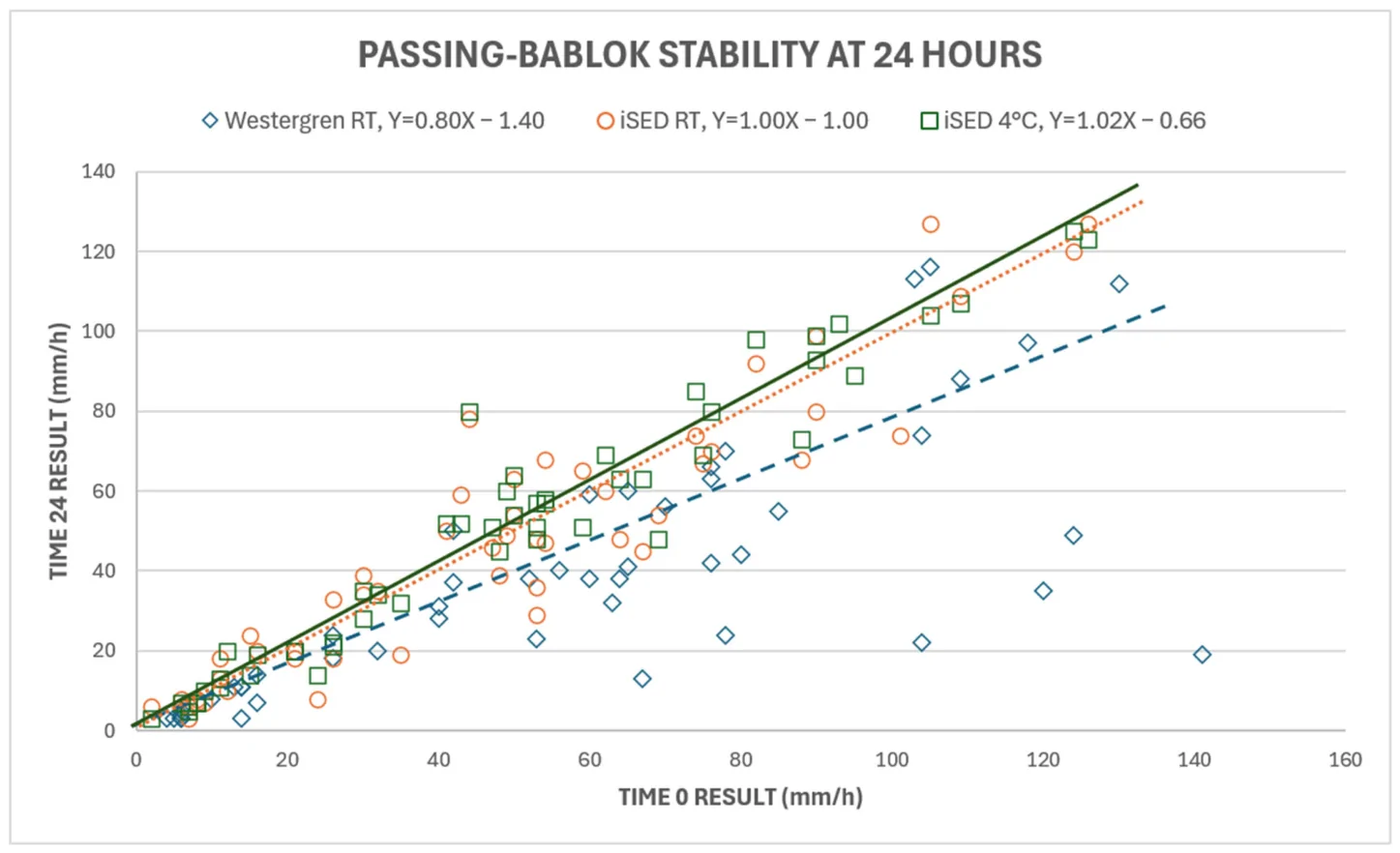
PassingBablok stability at 24 h. Individual Time 0 baseline data points are plotted against the results generated after 24 h from all three cohorts: Westergren at RT (blue diamonds/dashed line), iSED at RT (orange circles/dotted line) and iSED at 4 °C (green squares/solid line).

These data were visually analyzed, in part, by the Passing-Bablok data shown in Figure 1 where the individual Time 0 baseline data points are plotted against the results generated after 24 h from all three cohorts.

**Figure 2:**
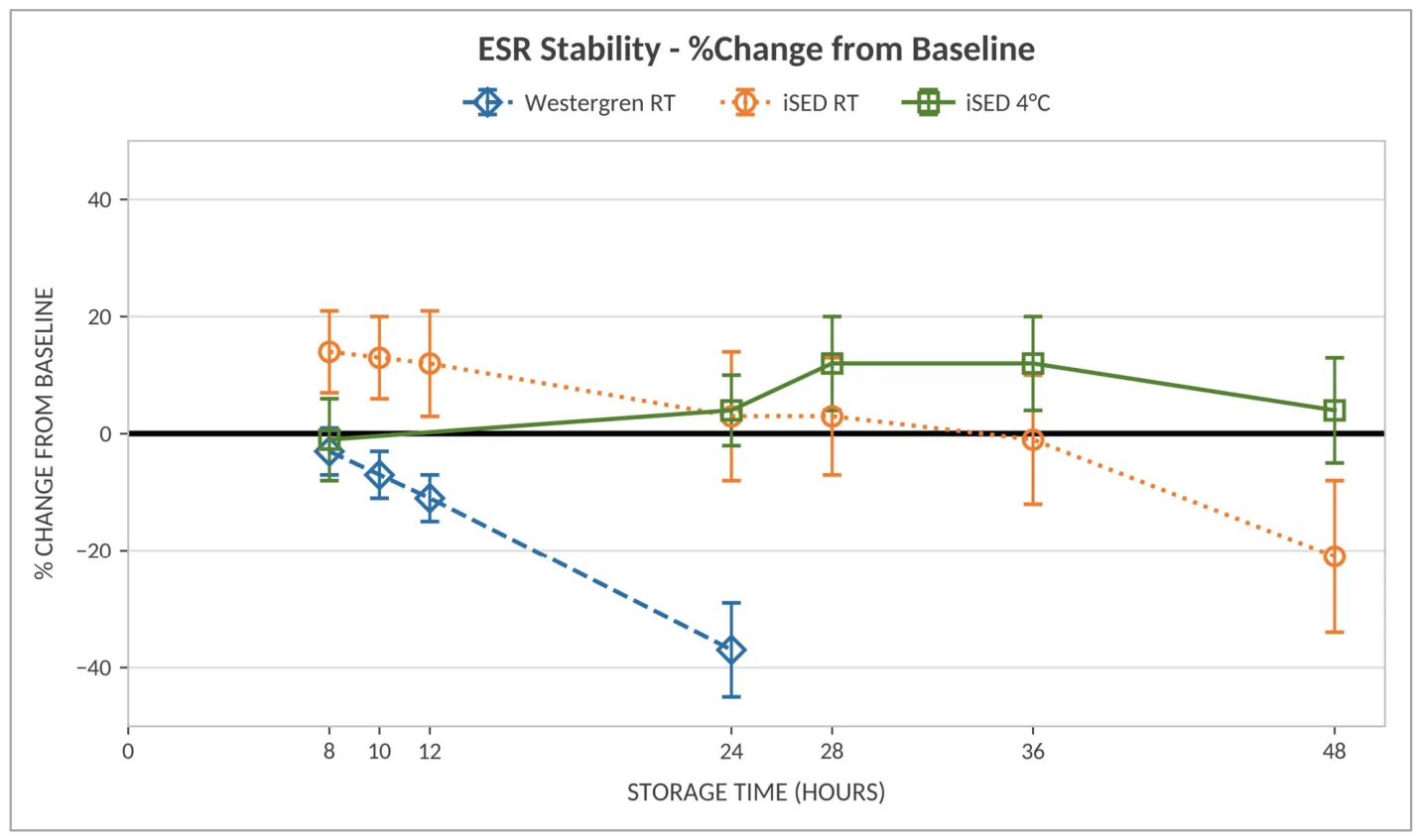
Sample Stability Drift plot. The % change between the mean ESR results from Time 0 and the other timepoints are plotted against the storage time. Westergren at RT (blue diamonds/dashed line), iSED at RT (orange circles/dotted line) and iSED at 4 °C (green squares/solid line).

The data were also evaluated qualitatively with a Stability Drift plot (Figure 2). The percent difference between the mean results a given time point (8, 10, 12, 24, 28, 36, and 48 h) and the baseline mean measurement are plotted against that time point in hours.

## 4. Discussion

Using the iSED method, ESR results were accurate for samples stored for up to 28 h at RT and up to 48 h at 4–8 °C. Sample stability up to 24–28 h at room temperature was initially observed during internal research and the Stabil-iSED study was commissioned to validate and quantitate the observation in a third-party laboratory. The results presented here confirms those initial observations in our initial evaluation studies, and we can therefore say with confidence that samples evaluated with iSED can maintain stability for 28 h when stored at room temperature and 48 h at 4–8 °C.

Samples tested with the Westergren method stored at room temperature were found to be stable at 8 h and 10 h. Degradation was already starting to occur around these timepoints as indicated by the 95% CIs of the slope (Table 3). These results are in alignment with other studies of sample stability of Westergren [4] and modified Westergren [26] methods.

The stability of samples was also qualitatively demonstrated by the 24 h Passing–Bablok analysis (Figure 1) and the extended Stability Drift analysis (Figure 2). In the Passing–Bablok analysis, the stability of the iSED cohorts is reflected by regression slopes that remain close to the line of identity (slope = 1.00) and y-intercepts that remain near 0.00. The Westergren cohort exhibited a regression slope of 0.80 at 24 h, indicating a deviation from the baseline measurements. In the Stability Drift plot, the mean percent difference from baseline remained relatively constant over time for each cohort until sample degradation created a progressive decline. This decline became apparent at 10–12 h in the Westergren/RT cohort and at 36 h in the iSED/RT cohort. There was no evidence of degradation in the iSED/4°C cohort through 48 h.

The use of Passing-Bablok correlation statistics as the acceptance criterion for stability is uncommon but, in the case of ESR, appropriate. ESR as a testing method does not have an established total allowable error (TEa). It was not included in the CLIA ’88 regulated list [27]. Use of an absolute bias acceptance criteria, such ±5 mm/h or ±10%, was considered and set aside due to the dynamic range of the assay (0–130 mm/h) and the low normal/abnormal cutoff (15–30 mm/h depending on age and gender). Specimens with high ESRs could vary by more than 5 mm/h or 10% with no clinical significance to that variance. As discussed earlier in this manuscript, ESR is a biological phenomenon versus the determination of the concentration of a defined analyte. For these reasons we selected novel approaches to two well established statistical methodologies; Passing-Bablok correlation statistics and Stability Drift analysis. For each sample cohort, the ESR values at defined time points were compared with the baseline value for that cohort. Acceptance limits were established as discussed earlier in this manuscript and are aligned with the acceptable correlation statistics discussed in the 2017 ICSH guidelines [23]. Results observed outside these limits were attributed to sample instability for that testing methodology and storage condition.

The iSED/RT and iSED/4–8 ° C results at the 8 h timepoint, where the y-intercepts did not meet the confidence interval acceptance criteria, warrants discussion. Upon closer examination, it was observed that the actual intercepts were within 2 mm/h of 0.00 (1.89 and −1.62, respectively) and the CIs were very narrow, each <4 mm/h in width. The clinical impact of these results is minimal. Examination of the raw T0 vs. T8 data for the iSED/RT cohort shows only two of 54 (3.7%) specimens that shifted from abnormal to normal (Table 4). In the iSED/4°C cohort the same two specimens and one additional specimen (three of 52; 5.7%) would have shifted from abnormal to normal (Table 4). It is important to recognize that, based on the nature of ESR, true instability does not resolve with time. More importantly, these cohorts met the stability criteria at several timepoints after 8 h. Therefore, we concluded that the 8 h sample stability results for iSED were acceptable.

Three uncharacteristic data shifts in the ESR values between the 12 h and 24 h timepoints were noted in the iSED/RT cohort. These were statistically identified as outliers and all data from these samples were excluded from the analysis in Table 3. If the outliers were included in a re-analysis, the iSED/RT data passed the acceptance criteria through the 12 h timepoint. The data from the 24 h and 28 h timepoints met the slope and intercept acceptance criteria but did not meet the correlation coefficient criteria (Table 5). Even with the outliers, the iSED/RT nearly passed the 24 h and 28 h timepoints, giving added confidence that the results with the outliers removed are valid.

We believe that sample stability was significantly longer with the iSED than the Westergren test because iSED technology is based on a rapid optical measurement technique and microfluidic technology that directly analyzes the aggregation of RBCs during the first few seconds of rouleaux formation in a controlled testing environment/flow cell and isolates it from time-sensitive effects that impact the Westergren method. The Westergren test differs by indirectly measuring ESR after the three phases of sedimentation [25], and it is subject to a range of known limitations including environmental, sample, and operator variables. In contrast, iSED testing is less dependent on the time-sensitive morphological changes in RBCs that can affect sedimentation. RBCs have been found to experience crenation and loss of central pallor after 8 h and 16 h when stored at room temperature [28]. We hypothesize these changs in RBC structure can have an impact on sedimentation and cell packing in the traditional Westergren method. iSED-based testing maintains its accuracy over extended periods, making it more robust and reliable for samples stored at room temperature.

The sample stability using photometric rheology is a significant advantage for clinical laboratories. In clinical practice, extended sample stability provides considerably more flexibility when storing and transporting samples, which can reduce the number of incorrect test results, the number of rejected samples, and the subsequent need for repeat phlebotomy. With longer stability, the same EDTA tubes can realistically be used for both ESR and CBC testing, and add-on ESR tests requests can be considered. The reduction or elimination of a dedicated ESR sample tube and an additional venipuncture will result in real cost savings for healthcare systems [29]. In addition, photometric rheology can eliminate off-label testing of samples stored for more than 4 h at RT. Finally, photometric rheology produces ESR results in <20 s and only requires 100 μL of whole blood that is sampled directly from the collection tube [24]. In contrast, the Westergren method requires 1000 μL, which must be pipetted from an uncapped collection tube and requires one hour to generate results [25]. Thus, time, effort, and biohazard exposure to laboratory personnel are minimized with photometric rheology.

Accurate, reproducible ESR results are important in patient care, especially when monitoring disease progression and response to treatment. While non-specific, ESR values can be used in scoring disease activity in conditions—such as rheumatoid arthritis [8], pediatric Crohn’s disease [30], polymyalgia rheumatica [31], and giant cell arteritis [32]—and age-related sample degradation could potentially impact how patients are classified.

The extended sample stability observed with the photometric method ensures dependable ESR values for up to 28 h at RT and 48 h refrigerated, resulting in values the laboratory and clinician can trust even when the sample has traveled from decentralized locations during which temperature excursions can occur or to minimize the need for additional sample draws when repeat or add-on testing is requested. Extended stability also affords the laboratory more time to wait before processing ESR samples—flexibility that cannot be taken for granted when lab resources are scarcer than ever [33,34] and samples may arrive during hours of minimal staffing. What is most important is the knowledge that the ESR values used to aid in the care of patients are accurate and have not been altered by sample age and degradation.

The current study has limitations. Low hematocrit levels are known to falsely elevate Westergren results. This is corrected by applying the Fabry formula to the Westergren results from patients with hematocrits below 35%. Hematocrit levels were not collected for the patients in this study. Had Fabry correction been applied to Westergren results from patients with hematocrit below 35%, the overall results from that cohort would have decreased, potentially reducing the sample stability conclusions. Another limitation is the asymmetry of sample testing, with the Westergren cohort being tested at 0, 8, 10, 12, and 24 h and only one storage condition, whereas the iSED RT and 4 °C cohorts were tested at 0, 8, 10, 12, 24, 28, 36, and 48 h. This scheme permitted the use of only one 7 mL blood draw for Westergren testing. To test the stability of a Westergren/4 °C cohort would have required drawing another 7 mL tube from the volunteers which was a patient safety concern. Although the sample size was based on ICSH recommendations for ESR test validation, it was still relatively small. Lastly, the effect(s) of additional factors or specific disease states (e.g., anemia of chronic disease, polycythemia vera, or sickle cell disease) that could negatively impact sample stability is not fully understood; thus, future studies should explore what factors might exist and might be relevant.

In summary, photometric rheology technology provides accurate ESR results for samples stored for up to 28 h at RT and up to 48 h at 4–8 °C. Samples tested with the Westergren method are stable for up to 10 h after collection when stored at RT.

## Figures and Tables

**Table 1:** Stabil-iSED Patient Demographics.

**Age (Years)**	**Mean**	**Median**	**Low**	**High**		
	52.54	54.5	25	82		
**Gender**	**Male**	**Proportion**	**Female**	**Proportion**		
	38	56%	30	44%		
**Ethnicity**	**White**	**Proportion**	**Af. Amer.**	**Proportion**	**Hisp/White**	**Proportion**
	44	65%	22	32%	2	3%

Af. Amer.: African American. Hisp/White: Hispanic or mixed Hispanic-White.

**Table 2:** Blood sample collection for each testing method and storage condition.

ESR	Westergren/RT Cohort, *n*	iSED/RT Cohort, *n*	iSED/4–8 °C Cohort, *n*
≤40 mm/h	21	21	21
41–79 mm/h	18	21	21
≥80 mm/h	13	12	10
Total	52	54	52

ESR, erythrocyte sedimentation rate; RT, room temperature.

**Table 3:** Blood sample stability *.

Timepoint (h)	Slope (95% CI)	*Y*-Axis Intercept (95% CI)	Spearman’s Correlation Coefficient
**Westergren/RT cohort**			
8	1.00 (0.95 to 1.02)	−1.00 (−1.93 to 0.52)	0.98
10	0.95 (0.90 to 1.00)	−0.69 (−2.0 to 0.80)	0.98
12	**0.92 (0.87 to 0.99) ^†^**	−0.60 (−2.46 to 0.55)	0.98
24	**0.80 (0.65 to 0.89) ^†^**	−1.40 (−5.95 to 1.27)	**0.79 ^†^**
**iSED/RT cohort**			
8	1.01 (0.96 to 1.07)	**1.89 (0.25 to 4.19) ^†^**	0.96
10	1.00 (0.93 to 1.10)	2.00 (−0.90 to 3.40)	0.96
12	1.00 (0.92 to 1.08)	2.00 (−0.27 to 4.31)	0.96
24	1.00 (0.90 to 1.12)	−1.00 (−3.44 to 3.07)	0.92
28	0.93 (0.84 to 1.05)	1.52 (−2.80 to 3.97)	0.90
36	0.93 (0.81 to 1.03)	1.43 (−2.63 to 5.30)	**0.88 ^†^**
48	0.85 (0.68 to 1.00)	−1.41 (−6.00 to 3.34)	**0.82 ^†^**
**iSED/4–8 °C cohort**			
8	1.07 (1.01 to 1.11)	**−** **1.62 (** **−** **3.94 to** **−** **0.33) ^†^**	0.96
24	1.02 (0.97 to 1.09)	−0.66 (−1.92 to 1.23)	0.96
28	1.02 (0.96 to 1.11)	1.13 (−1.51 to 3.42)	0.96
36	1.00 (0.92 to 1.13)	2.00 (−6.83 to 3.10)	0.95
48	0.94 (0.85 to 1.03)	1.32 (−1.54 to 3.46)	0.95

CI, confidence interval; RT, room temperature. * The results of a given timepoint and testing condition were plotted against the respective baseline timepoint (time 0) and correlation statistics were determined by the Passing-Bablok regression analysis. Samples were considered to be stable if the 95% CI of the slope included 1.00, the 95% CI of the *y*-axis intercept included 0.00, and the Spearman’s correlation coefficient was ≥0.90. ^†^ The data did not meet the acceptance criteria for this parameter at this timepoint.

**Table 4:** Specimens with ESR results that shifted from abnormal to normal.

		iSED/RT Result (mm/h)		iSED/4° C Result (mm/h)	
Specimen	Normal Range (mm/h)	T0	T8	T0	T8
30 y.o. F	<20 mm/h	24	14	24	11
40 y.o. F	<20 mm/h	21	19	21	19
67 y.o. F	<30 mm/h	NA	NA	30	20

y.o: years old. F: Female.

**Table 5:** Passing-Bablok analysis of iSED/RT data with outliers included.

Timepoint (h)	Slope (95% CI)	*Y*-Axis Intercept (95% CI)	Spearman’s Correlation Coefficient
iSED/RT cohort			
8	1.00 (0.94–1.05)	2.00 (0.63–3.93)	0.97
10	0.98 (0.92–1.06)	2.16 (0.29–4.33)	0.96
12	0.98 (0.91–1.04)	2.26 (0.00–4.55)	0.96
24	0.97 (0.85–1.07)	0.00 (−4.24–3.67)	**0.86 ^†^**
28	0.89 (0.78–1.00)	1.92 (−2.50–5.76)	**0.85 ^†^**

CI, confidence interval; RT, room temperature. * The results of a given timepoint and testing condition were plotted against the respective baseline timepoint (time 0) and correlation statistics were determined by the Passing-Bablok regression analysis. Samples were considered to be stable if the 95% CI of the slope included 1.00, the 95% CI of the *y*-axis intercept included 0.00, and the Spearman’s correlation coefficient was ≥0.90. ^†^ The data did not meet the acceptance criteria for this parameter at this timepoint.

## Data Availability

The data that support the findings of this study are available from the corresponding author upon reasonable request.

## Abbreviations

The following abbreviations are used in this manuscript:

CFR	Code of Federal Regulations
CI	Confidence interval(s)
EDTA	Ethylenediaminetetraacetic acid
ESR	Erythrocyte sedimentation rate
FDA	Food and Drug Administration
h	Hour(s)
ICSH	International Council for Standardization in Hematology
IRB	Institutional Review Board
min	Minute(s)
*n*	Sample size
RBC	Red blood cell(s)
RT	Room temperature
s	Second(s)
